# Aspects of Data Ethics in a Changing World: Where Are We Now?

**DOI:** 10.1089/big.2018.0083

**Published:** 2018-09-17

**Authors:** David J. Hand

**Affiliations:** Department of Mathematics, Imperial College, London, United Kingdom.

**Keywords:** algorithms, confidentiality, data ownership, personal data, privacy, trustworthiness

## Abstract

Ready data availability, cheap storage capacity, and powerful tools for extracting information from data have the potential to significantly enhance the human condition. However, as with all advanced technologies, this comes with the potential for misuse. Ethical oversight and constraints are needed to ensure that an appropriate balance is reached. Ethical issues involving data may be more challenging than the ethical challenges of some other advanced technologies partly because data and data science are ubiquitous, having the potential to impact all aspects of life, and partly because of their intrinsic complexity. We explore the nature of data, personal data, data ownership, consent and purpose of use, trustworthiness of data as well as of algorithms and of those using the data, and matters of privacy and confidentiality. A checklist is given of topics that need to be considered.

## Introduction

The automation of measurement and data collection procedures, coupled with the development of vast capacity for data storage and the creation of highly sophisticated tools for analyzing and processing data, often in real time, is radically changing the world in which we live. This has prompted considerable debate, both philosophical and legal, about the *right*, *legitimate*, and *proper* ways to use such data. Also, since there is no absolute authority to whom we can appeal for guidance, it is important that we, the data creators, suppliers, and users, should engage with these ethical considerations.

Emphasis naturally tends to be on the side of risk and protection, but we must always bear in mind the need for a proper balance between risk and benefit. Zero risk can be attained only at the cost of zero benefit, but the potential benefit from new data technologies is vast. Or, as leading data ethicists Floridi and Taddeo^1^ put it: “On the one hand, overlooking ethical issues may prompt negative impact and social rejection …On the other hand, overemphasizing the protection of individual rights in the wrong contexts may lead to regulations that are too rigid, and this in turn can cripple the chances to harness the social value of data science.”

We must also recognize that we cannot expect to give simple answers to complex moral problems involving data. This will often be impossible, not least because the data environment is changing so rapidly. Instead, the aim must be to help focus on the issues and attempt to remove confusion and ambiguity, so as to provide *principles* that can help people come to a conclusion about what is the right way to act and the right thing to do in the circumstances in which they find themselves.

Furthermore, in considering ethical matters, we must consider both current and future uses of data. Progress in data science and technology is often described as if it were a question of reaching a new status quo: as if, once we have developed and implemented tools for handling the vast data sets and real-time issues, we can relax. However, that is to misunderstand the nature of the changes we are witnessing. The changes are and will be ongoing. We are not approaching a plateau but are on the slopes of doubtless even more dramatic changes. This will occur through the application and implementation of existing tools, and also through the creation of new data technologies, with current examples being blockchain, homomorphic computation, and quantum computing.

The ubiquity of the impact of data technologies on all aspects of modern life means that those concerned with data and their use must engage with the ethical issues. This imperative represents something of a qualitative change: domains such as statistics and computer science, which are at the center of the data revolution, have previously focused largely on the technical matters. However, the acceptability of the tools now being developed and the ways they are being used is highly sensitive to subtle ethical issues. We are seeing this forcefully at the time of writing with something of a backlash against the freedom of social media companies, which up until now have been relatively unconstrained in what they do and how they do it.^2,3^

Matters would be comparatively straightforward if the ethical issues presented by new data technologies replicated those of other technical and scientific areas, but they often present novel challenges of their own. Here are two examples.

In the past, most analyses were based on static collections of data accumulated through painstaking manual measurement procedures. Increasingly, however, automatic measurement procedures mean not only that massive data sets are painlessly and cheaply accumulating but also that the measurement is ongoing. This opens the scope for real-time and online analysis and decision-making—and to recommender systems, autonomous vehicles, in-journey travel route optimization, predictive policing, and so on. Also, this in turn opens up the possibility of intertwining analyses aimed at research with those aimed at operations. This has always been the case to some extent—for example, in late-stage clinical trials or in the use of gold samples in credit scoring—but the scope is now much greater. A familiar example is the use of two-arm experimental designs (A/B or “champion/challenger” studies) to optimize things such as advertising websites, and a controversial recent example is the Facebook emotional contagion study.^4^ The distinction between the two types of analysis—those aimed at research and those aimed at practice—was noted in the 1979 Belmont report on ethical principles for the protection of human research subjects, which commented that “it is important to distinguish between biomedical and behavioural research, on the one hand, and practice of accepted therapy on the other, in order to know what activities ought to undergo review for the protection of human subjects of research.”^5^ More specifically, if data are not just simply collected blindly as exhaust from some operation but are also influenced by active intervention in that operation, then careful thought is needed about the ethical aspects of data collection. Metcalf and Crawford^6^ present an illuminating discussion of the research/practice distinction, and its validity in the modern data context.

A second example involves the 1991 U.S. Federal Policy for the Protection of Human Subjects, the so-called Common Rule.^6,7^ This is a U.S. ethical structure for biomedical and behavioral research on human subjects, based largely on the Belmont report. The Common Rule,^7^ Section 46.101(b), gives a number of exemptions from these ethical guidelines. Among these exemptions is “Research involving the collection or study of existing data, documents, records, pathological specimens, or diagnostic specimens, if these sources are publicly available or if the information is recorded by the investigator in such a manner that subjects cannot be identified, directly or through identifiers linked to the subjects.” Now, one of the characteristics of modern data work is that very often analyses are made on existing publicly available data sets (often characterized as “open data”), and it is one of the particular strengths of these new technologies that discoveries can be made by linking and merging them. Such discoveries can be of scientific and medical value (e.g., in epidemiology), of economic value (e.g., in estimates of gross domestic product or inflation), and they can also be highly sensitive. There are, for example, classic cases of individuals being reidentified by linking public domain anonymized data sets. At the very least, this involves invasion of privacy, and it can lead to worse.

A fundamental aspect of this is that one does not know, indeed *cannot* know, how data will be used in the future, or what other data they will be linked with. This means we cannot usefully characterize data sets as public (vs. not public) or by potential use (since these are unlimited and unforeseeable), and that the intrinsic nature of the data cannot be used as an argument that they are not risky. It is not the data *per se* that raise ethical issues, but the use to which they are put and the analysis to which they are subjected.

In summary, various properties of modern data and the use of such data make data technology distinct from other advanced technologies, requiring careful consideration of ethical issues. These include the following:

- The pervasiveness of modern data technology means we might legitimately regard it as an aspect of societal *infrastructure*, in the same way that mathematics, language, transport, and so on are infrastructural.
- The interconnectedness of data. Data on travel may be used for discovering things far beyond mere travel patterns; data on purchases are not solely relevant to purchases; and so on.
- The dynamic nature of data. Modern data sets often evolve and accumulate over time so that they may permit discoveries in the future that they do not permit today.
- Real-time and online analysis and decision-making, as data arrive.
- Synergistic analysis through merging and combination of data sets.
- Lack of space, time, and social context limitation on scope of data (data may describe and be used regardless of where, when, and for what purpose they were collected).
- Ability to use for unexpected purposes and to reveal unexpected information (this is the core purpose of data mining).
- Risk of exceptional intrusiveness since it is impossible to avoid having data about individuals stored in multiple databases.
- Potential for misuse, privacy breach, blackmail, and other crimes.
- Subtle ownership issues (“my” data might also be your data; I can sell “my” data while retaining them, and so on, as discussed in detail below).

All of these issues, and others, can present novel ethical challenges.

Ethical principles provide a broad and high-level context for resolving ethical dilemmas. For general ethics codes, Metcalf^8^ lists nine inward facing and seven outward facing purposes. For us, the outward facing ones are the most relevant. These are the following:

- protect vulnerable populations who could be harmed by the profession's activities;
- protect/enhance the good reputation of and trust for the profession;
- establish the profession as a distinct moral community worthy of autonomy from external control and regulation;
- provide a basis for public expectations and evaluation of the profession;
- serve as a basis for adjudicating disputes among members of the profession and between members and nonmembers;
- create institutions resilient in the face of external pressures; and
- respond to past harms done by the profession.

Note that these purposes are couched in terms of “professions.” Professions typically have their own formal code of practice based on ethical principles, but one of the characteristics of modern data science and technology is that there is no unique profession bearing responsibility for it. Admittedly, statistics and computer science are the two core disciplines, but others, not least a large range of application disciplines, also have a presence. Different application domains (e.g., medicine, social media, retail, and finance) will map the principles to practical ethical guidelines in different ways.

As far as ethical codes for data collection, manipulation, and use are concerned, these have various functions, including things such as the following:

- providing guidance on how to behave in difficult circumstances;
- preserving privacy in a way that users and the public will find acceptable;
- ensuring that data are used in such a way as to benefit the public;
- reassuring customers, the public, and others about an organization's integrity; and
- reassuring employees that they work for a trustworthy organization.

As might be obvious from this list, sometimes there is a tension between these desiderata. A familiar example is the use of large-scale collated medical records to infer conclusions about disease progression and effective treatment. Clearly this is to the benefit of the public, but equally clearly the data describe individuals, so that their data must be divulged at some level: their privacy may be at risk.

Things are further complicated by the fact that the public's view of such matters is heterogeneous, varying not only between groups of people but also over time—often in response to publicity and news reports. Views also change (generally in a positive way) with growing understanding of the value of data and the way the data can be used.

In an ideal world, we would be able to come up with precise guidelines on how one should behave in different circumstances. However, the context of data science is so vast and diverse, and is changing so rapidly over time, that we cannot hope to put in place precise regulations. There cannot be a single and simple universal set of rules, and unexpected and unforeseen circumstances are certain to arise. The best we can hope for are some ethical principles that have to be interpreted or instantiated in particular applications. That is, the principles must be mapped to low-level guidance, and this is likely to be application specific.

The principle-based approach is a common strategy in such changing environments. Examples in the data world include the original *Code of Practice* of the UK Statistics Authority based on eight principles, the report of the joint British Academy/Royal Society on *Data Management and Use: Governance in the 21st Century* based on five principles, the Accenture *Universal Principles of Data Ethics* that had twelve principles, the *ACM Code of Ethics and Professional Conduct* beginning with seven general moral principles (which “a computing professional should …”), and others.

At the highest level, the principles include such things as integrity, honesty, objectivity, responsibility, trustworthiness, impartiality, nondiscrimination, transparency, accountability, fairness, robustness, resilience, usability, efficiency, and independence. All good and desirable characteristics. These are then refined into lower, but still high-level principles. For example, for data science in government, Drew^9^ suggests six main principles:

- start with clear user need and public benefit;
- use data and tools that have the minimum intrusion necessary (“data minimization”);
- create robust data science models (e.g., to avoid improper discrimination);
- be alert to public perceptions;
- be as open and accountable as possible; and
- keep data secure.

While one might hope to anticipate future data-related challenges, the fact is that this will be very difficult. In particular, the law is generally changed in response to issues that have arisen, rather than those that will (or might) arise: legislation is typically retrospective. Law provides a set of regulations that members of a society must adhere to, at pain of incurring penalties. In contrast, ethics guide people's conduct, to help them do what is morally right. However, if ethics guide what a person *should* do, while law sets down what they *must* do, they are obviously linked.

The remainder of this article is structured as follows. The next section describes modern data, their genesis, and the perception that they are a new resource, even a new commodity. This is followed by an examination of personal data, the concept underlying the General Data Protection Regulation (GDPR). The notion of data ownership is a difficult one, since the nature of data is such that they are often not clear who owns them, or indeed if they can be owned. This is examined in the Data Ownership section. The Consent and Purpose section examines two aspects, which are often seen as the core facilitating issues in guiding the ethical use of data. In fact, however, underpinning all of this are notions of trustworthiness, examined in the Trustworthiness section. Privacy and confidentiality are seen as one side of the risk/reward balance, but as we see in the Privacy and Confidentiality section, the notion of privacy in a data context is rather nuanced. The web, for example, both enhances and detracts from personal privacy. The final section, Conclusion, pulls things together, to yield a list of recommendations for tackling the confusing and complex face of practical data ethics.

## Data

Data are often described as the new coal or oil. This is on the basis that coal and oil were the fuels that drove the industrial revolution, and data are seen as serving a corresponding role for the information revolution. Like coal and oil, data are processed to extract value. However, there are fundamental differences, so the usefulness of the metaphor is limited. In particular, while coal and oil are *consumed* when extracting value (in the form of energy), data are not consumed when they are processed to extract value. Data can be reused any number of times without being consumed or diminished. Data can even be shared or sold, without the original possessor relinquishing it. Data can be used in multiple independent, even unsuspected ways, ways that may not initially be apparent and may only become so in the future, perhaps when data sets are combined. In general, data cannot be depleted, although their value for some particular purpose can be reduced. Furthermore, since the value of data depends on the context, so also will the price—also unlike fossil fuels.

In fact, one might go further than this and say that data exist only in a context. Certainly, an isolated number is not a datum. Numbers become data only when additional metadata are provided—at a minimum the unit of measurement must be specified. Specifying a unit of measurement is equivalent to making a comparison between objects,^10^ putting one object in the context of another. Of course, one can go further still and question whether data can make sense or be interpreted outside the context of some theory or worldview, but exploring that would take us too far from the matters of direct concern to this article.

In any case, the central concern of this article is not data *per se*, but how the data are used. In particular, different uses arise from bringing different data items together—making different comparisons, noting the common identity of an object underlying two different data items, aggregating data to draw general conclusions, and so on. Also, there are an unlimited number of ways in which this may be done.

We have already remarked that one of the most important drivers behind the impact of modern data technology on society is that the data are often (not always, as we shall see) captured automatically, during the course of some activity. This means, at least in principle, that no additional effort or resource is needed to accumulate massive data sets, which can subsequently be analyzed (for an extensive discussion of such “administrative data” see Hand,^11^ and the associated comments). An important aspect of such data is that they are “observational.” They have not been deliberately collected following some intervention, as in a designed experiment, which means they are at risk of unsuspected biases and distortions. Furthermore, “In a real sense, administrative data often tell us what people *are* and what they *do*, not what they *say* they are and what they *claim* to do. We might thus argue that such data get us closer to social reality than do survey data,”^11^ and as such, administrative data, “which record actual activity, may be very different from what we put on Facebook of Twitter.”^12^ Indeed, there has been publicity recently about the depressive effects of social media because users tend to present a positive face on such media, leading to the impression that in contrast to the wonderful lives everyone else is apparently leading, yours is not so good.

The advent of automatic data capture also has other implications. In the past, collecting data required effort and resources, so they were collected only if someone had a use for them. However, since data can now be captured essentially effortlessly, the attitude is often “if it might be useful, record and store it.” The prices of goods in a customer's shopping trolley are noted so that the bill can be calculated—but then the prices can be effortlessly stored in a database. This can sometimes lead to disappointment: merely because having a vast body of data does not mean they are necessarily of any value for answering particular questions you have in mind.

The practice of recording everything, partly because the data might be useful in the future and also partly because they are now so cheap to record, is contrary to the data minimization principle: that one should store only those data that are necessary for some purpose (defined in GDPR Chapter II Article 5 as personal data that are “adequate, relevant, and limited to what is necessary in relation to the purposes for which they are processed”). It represents a fundamentally different attitude: possibility driven rather than purpose driven. This poses an important tension between the perceived potential of data and data science for improving the human condition, and the desire to preserve the privacy of individuals described by the data.

The low cost of accumulating large masses of data means we, as individuals, cast long data shadows. These are the data traces that result from ordinary daily activity, such as using a credit card, a travel card, accessing social media, web searching, e-mails, making phone calls, and even interacting with an electronic “intelligent personal assistant” such as Amazon's Alexa. Such traces reveal huge amounts about what people get up to, who they interact with, what their interests are, and even what their beliefs are. They bring into sharp focus some of the ethical issues: the ability to trawl someone's data shadow is invaluable in tracking terrorists and criminals (indeed, terrorists have been tracked down by following their movements as determined from mobile phone masts), but on the other hand, this ability is also invaluable in tracking law-abiding members of society who hold views that an authoritarian government may dislike, as well as leading to embarrassment and even blackmail if they fall into the wrong hands. Unlike real shadows, data shadows may linger for a long time—causing potential embarrassment and worse far into the future.

## Personal Data

Inevitably, most of the ethical issues of data relate to data about people. The GDPR came into effect on May 25, 2018. This replaces the UK's Data Protection Act (DPA), and is a European Union (EU)-wide regulation “on the protection of natural persons with regard to the processing of personal data…,” defining personal data as “any information relating to an identified or identifiable natural person (‘data subject’); an identifiable natural person is one who can be identified, directly or indirectly, in particular by reference to an identifier such as a name, an identification number, location data, an online identifier or to one or more factors specific to the physical, physiological, genetic, mental, economic, cultural or social identity of that natural person” (GDPR Article 4, Definitions^13^). This includes things such as IP addresses and mobile device identifiers. Even pseudonymized personal data might be covered by the regulation. The regulation increases the level of responsibility and accountability of those processing personal data, and requires organizations to notify the national authorities of serious data breaches as soon as possible—within 24 hours if feasible. It also gives greater control to individuals, who will have easier access to “their own” data (we return to the question of data ownership below), be able to transfer personal data between service providers more easily (to improve competition), and have a “right to be forgotten,” so that they can request their data to be deleted if there are no legitimate grounds for holding them. Penalties for organizations breaching the regulation can be as much as four percent of global turnover.

The GDPR goes into considerable detail about the obligations associated with personal data and what legitimate operations may be applied to such data, although there will doubtless be many debates about the precise interpretation as it comes into force. Thus, Recital (1) says “The protection of natural persons in relation to the processing of personal data is a fundamental right …” but Recital (4) says “The right to the protection of personal data is not an absolute right,” going on to add that “it must be considered in relation to its function in society and be balanced against other fundamental rights, in accordance with the principle of proportionality….” Recital (4) also says “The processing of personal data should be designed to serve mankind….”

The UK's Information Commissioner's Office has published guidelines to determining what are personal data.^14,15^ These describe a sequence of steps (in the form of questions) that can be gone through to determine whether data are personal data. They hinge around whether an individual can be identified from the data, perhaps by linking to other data sources.

As we have noted, complications arise because data items do not exist in isolation, but only in a context. In particular, my personal data might tell you something about others' personal data, even if that is not explicit in a database. For example, my genetic data tell me something about other members of my family, and my address, holiday times, and so on are likely to describe other people as well. Your personal data might include the fact that you belong to a particular group. However, the fact that that group met at a certain time and place is of itself surely not your personal data. However, put these facts together and I can deduce where you were then. Combine your personal data with other data and we have more personal data about you.

## Data Ownership

GDPR Recital (7) says “…Natural persons should have control of their own personal data…,” with a concept of ownership implicit. Before the computer and Internet era, discussions of data ownership were often based on notions of copyright. Indeed, data sets were sometimes protected against copying by introducing unique features. For example, mathematical tables might include the occasional wrong value in the 4th (insignificant) digit and maps might include imaginary towns or nonexistent kinks in roads. More recently, “digital watermarking” is sometimes used, in which an identifying signal is embedded in the data. This is related to steganography, where a message or other information is concealed in a body of data.

This is all very well if it is clear who owns a given body of data. However, unfortunately, in the context of data, the concept of ownership is often ill-defined. In contexts other than data, something is owned by someone (or more generally some entity such as a corporation or public body, but for convenience we will refer to a person here) if that person has the right to control how the thing is used. More formally, this means the owner has legal title and full property rights, including the right to profit from the use. However, that only works if notions of legal title and the sense in which data are property are clear. We are in danger of circular definitions.

Also, in other contexts, the creator or legal entity for whom the creator is working is typically the initial owner. A mining company (having acquired the right to mine) will be the owner of the coal it digs up. A software company will be the owner of the code its employees write. A furniture manufacturer will own the chairs it produces. However, what about personal data, data “about” someone? While someone else might go to the trouble of “creating” data describing my height and weight, these measurements are about me. If a shop records the prices of the goods you have bought, so it can calculate how much to charge you, who owns those data? More currently, should Google be regarded as owning search histories, each track made by an individual user, and each personal data? Should Amazon be regarded as owning data on behavior relating to individual customers?

The critical aspect of this is that the data are personal—are identifiable—and various authors (e.g., Greenwood et al.,^12(p.207)^) take the perspective “that ownership of personal data rests with the people that data are about.” If the data are anonymized, with interest lying solely in aggregate aspects, then at least in principle no problems arise. If a psychologist measures characteristics of a hundred people, deleting information that might allow them to be reidentified, then the data will be regarded as belonging to the researcher. It is the researcher's efforts that have provided the value that the data possess. Incidentally, this value has been the subject of heated debates in the context of open data (“data that can be freely used, reused, and redistributed by any one subject only, at most, to the requirement to attribute and share alike,” http://opendefinition.org/), especially research data, which a journal might require to be made accessible on the web as a precondition for publication of an article analyzing those data. Someone who has spent substantial time and effort in collecting data might be unwilling to reveal the data to so-called research parasites who can ride on the coattails of their efforts. In other areas—astronomy for example—where the data collection is clearly a large team effort, data are willingly shared via public databases.

In 2007, Alex Pentland in 2007 suggested to the World Economic Forum^16^ that individuals might have control over their personal data, so that “a person's data would be equivalent to their ‘money.’ It would reside in an account where it would be controlled, managed, exchanged, and accounted for just like personal banking services operate today.” I think this is idealistic but am not sure that it is realistic in terms of capitalizing on the potential of data for progress. Although “money” is quite an elaborate concept, at base it is a mechanism for facilitating exchange so that, by definition, money has the same value whatever it is used for. In contrast, the opposite is true of data, where the value depends on the context and use.

Moreover, with automatic data capture and the imminent Internet of Things, it is questionable whether attributing individual ownership of each data item is feasible—we have already commented about how data about one person imply information about other people. Also, further complications arise from derived data, in which the analysis and merger of data sets allow the deduction of more information about individuals, which would presumably need to be retained in individuals' data accounts.

With my technical statistician hat on, I am also very much aware that allowing people to refuse access is a sure way to distort data sets and produce misleading conclusions.

An alternative strategy might be that of “data commons”: trusted organizations that store data from a variety of bodies. There is something of an analogy with credit bureaus, which take data from multiple financial bodies, combine and analyze the data, and feed back the results of the analysis (e.g., in the form of credit scores). One advantage here is that the range of bodies feeding in data reduces selection bias risks.

This sort of approach has been explored on a smaller scale by various national bodies. The Administrative Data Research Network (ADRN)^17^ is one example. This links data from different UK government departments and other bodies in a secure environment, using multiple layers of protection, both technical and legal. Accredited researchers work on the data in approved projects for scientific and public policy projects.

Inevitably, the legal aspects of data ownership are also complex—interestingly, the GDPR does not mention “data ownership.” The EU Inception Impact Assessment on *European Free Flow of Data Initiative Within the Digital Single Market*^18^ drew attention to the legal uncertainty about data ownership, observing that “personal data cannot be ‘owned’ in the EU, but strict rules on access and use by anyone other than the person to whom the data refer are in place,” and going on to note that “a gap exists with regard to ‘ownership’ of nonpersonal data, particularly nonpersonal data that are machine generated.”

van Asbroeck et al.^19^ have reviewed the legal aspects in the EU, commenting that “the current legal framework relating to data ownership is not satisfactory…. No specific ownership right subsists in data and the existing data-related rights do not respond sufficiently or adequately to the needs of the actors in the data value cycle.” As one might expect, one complication is the lack of legal harmonization across jurisdictions. They also point out that “the issue of data ownership is even more complicated by the data value cycle, which can be rather complex and involves numerous stakeholders. This increases the difficulties in determining who could or would be entitled to claim ownership in data. Many of such stakeholders may attempt claiming ownership in data because, for instance, they create or generate data, or because they use, compile, select, structure, reformat, enrich, analyze purchase of, take a license on, or add value to the data.” So it is not simply the effort of collecting the data in the first place, which might confer ownership, but the effort of manipulating as well.

In more detail, among the conclusions reached by van Asbroeck et al.^19^ are the following:

- case law at EU level does not explicitly recognize an ownership right in data;
- that personal data are not necessarily owned by the individual, so that an ownership right for data controllers or processors (as defined in the GDPR) cannot be excluded, but that this would be subject to the individual's control over his or her personal data;
- the principles relating to ownership of physical entities are not always relevant to data;
- there are many regulations that may impact “a company's control of, the access to, or the rights in data”; and
- none of intellectual property rights or trade secrets provides “adequate protection of (ownership in) data.”

Suggesting that resolving the issues with contractual agreements would be burdensome and probably impossible to regulate with legal certainty, they go on to propose a solution via “the creation of a nonexclusive, flexible, and extensible ownership right in data(sets), with a data traceability obligation as a safeguard.” This notion of *traceability* seems to me to be fundamental to easing many of the ethical and nontechnical data challenges.

## Consent and Purpose

The notions of consent or informed consent have long been important in research, especially in healthcare research. In that domain, the idea is that consent should be obtained before an intervention, and that the intervention should be based on a sound understanding of its implications and possible consequences. However, it is questionable how much this is relevant to or practicable in the modern data world, for reasons including the two points noted above: that the very essence of the promise of modern big data is that future applications are unspecified and unknown (indeed, unknowable), and that the data typically already exist in databases so that studies are mostly noninterventional.

GDPR Recital (40) says that in order for processing to be lawful, personal data should be processed on the basis of the consent of the data subject concerned or some other legitimate basis, laid down by law. However, Recital (54) says that the processing of special categories of personal data may be necessary for reasons of public interest in the areas of public health without consent of the data subject (and see Article 6 for details). This is all very well, but Metcalf et al.^20^ noted that “The debates about how to handle consent in big data research have generated a remarkable diversity of positions, ranging from jettisoning informed consent altogether in noninterventional research that makes use of already existing or passively collected data sets, to calls to develop statistical methods and research infrastructures … to accommodate more dynamic notions of consent.”

In general, to be meaningful, informed consent to the use of data requires two conditions: (1) an understanding of *what* the data might be used for in the future and (2) an understanding of *how* the data are to be used.

The first of these is difficult because, as mentioned above, the future use is unknown. Moreover, the data might be merged with other data sets to reveal valuable information contained in neither alone: it might not be possible to say what use any given data set will contribute to. Merging of data sets is often valuable for exploring aggregate properties of a population—for example, whether two variables are related, when they are obtained from two different sources. However, merging can also be important for decisions relating to individuals. It is the essence of a great many analyses that they combine the data from an individual with summary data from a population of individuals, to make decisions. For example, clinical trials aggregate data from the trial subjects and the conclusions are then used to make decisions about treatments and doses for individuals.^21^

Recital (33) of the GDPR makes an attempt to tackle this intrinsic unknowability of future use: “It is often not possible to fully identify the purpose of personal data processing for scientific research purposes at the time of data collection. Therefore, data subjects should be allowed to give their consent to certain areas of scientific research when in keeping with recognised ethical standards for scientific research. Data subjects should have the opportunity to give their consent only to certain areas of research or parts of research projects to the extent allowed by the intended purpose.”

That is specific to scientific research, but one might try to specify a similar condition for other domains. For example, one could say that data might be used solely for the purpose of calculating credit scores, or solely for the purpose of identifying products one might be interested in, without going into minute detail. However, this does put the potential benefits of the data revolution at risk. Taking credit scoring as an example, aspects of behavior, residing in databases far removed from those currently stored by financial institutions, are increasingly being used in this context. One example is social media data: a number of start-ups have recently been launched using social media data to contribute to credit scores. The list of potential areas in which any given data might be used is vast. Expecting people to list such areas seems infeasible, short of using very high-level blanket categories.

Condition (2) above assumes that the person being asked to consent has the expertise and knowledge to understand how the data will be used. This will clearly be difficult. It is almost a defining characteristic of modern data analytic tools, such as neural networks, support vector machines, and ensemble systems, that they are intrinsically complex and defy simple explanation. And yet, the GDPR says that a data subject has a right to access “meaningful information about the logic involved,” and Recital 71 says data subjects have a right to “obtain an explanation of the decision reached after such assessment.” This poses a challenge. One strategy might be along the lines described in Hand and Yu.^22^ This is aimed at the long-standing legal requirement to provide an explanation to people seeking an explanation for why they have been declined credit. Since the models underlying credit granting decisions can be complex—neural networks, logistic regression trees, and so on, often involving hundreds of variables—explaining the underlying principles of the models and their practical implications for any particular individual is out of the question. Instead, Hand and Yu^22^ suggested a strategy for identifying which variables were important in reaching a decision, based on comparing models with and without each variable. Such a strategy could certainly be used to give “an explanation of the decision reached after such assessment.”

Data sharing is vital if we are to see the benefits of modern data technology. However, as we have seen, obtaining consent to such sharing might prove difficult. Much of the publicity associated with data sharing is concerned with individuals' concern about loss of privacy through the combination of data sets. However, there is also another side to this. This is that organizations might be loath to share their data. This can obviously be the case if the organization's business model is built on the value of the data, but it can also arise from simple caution. For example, the ADRN encountered resistance in its attempt to extract data sets for social science and public policy research: “However, during the last year getting access to data from government departments and providing it to researchers in a timely manner has presented the largest challenge to the Network. … It is apparent that within some departments there is a cultural reluctance to share data, with even those government departments who supported the Taskforce recommendations being reluctant to share data collected for operational purposes with the ADRN for independent high quality research.”^17^

This caution is understandable: data sharing comes with risk, and the press gleefully reports cases of data leakage or theft. Legislation, such as the Digital Economy Act, introduced since the above report, should help to ease such concerns.

Informed consent in research is a step on the way to ensuring the three pillars of *accountability* (the duty to justify an action and be answerable for its consequences), *transparency* (that actions should be done without secrecy, and be subject to examination), and *responsibility* (the obligation to ensure that an action is performed properly).

Although much of the debate about data ethics hinges on notions of responsible use of data in terms of constraints on what may be done with them, there is a complementary aspect of responsibility: the duty to act if an analysis detects abuse. Child sexual abuse rings and serial killers can often be detected earlier than would otherwise be the case through judicious analysis of extensive data sets, which might easily go beyond any analysis for which prior consent was given. A case might be argued that certain bodies, under certain circumstances, should be able to access wide-ranging data sets. The analogy with search and wiretapping is immediate. And as with those, rigid enforcement of strict codes of conduct and the following of due process will be necessary.

Finally, if informed consent is possible at all in the context of big data, it cannot be obtained by default: it has to be active, not passive. And yet, up until now, such passive consent is common—by default acquiescence to terms and conditions for products and services, without reading them. There are sound reasons that people do this—life is too short to plough through pages of tedious legalese trying to interpret what it might mean in practice, even assuming one had the technical knowledge to be able to understand it anyway.

## Trustworthiness

Some years ago, I described the use of statistical methods in pharmaceutical research to a lay audience. A woman in the audience said she was suspicious of all such research. After all, she pointed out, why would anyone sponsor research unless they had a vested interest in the outcome. This danger that research is used merely to support a particular position is indeed a very real one. Many case studies, from the pharmaceutical industry, the tobacco industry, the automotive industry, and beyond, have demonstrated this. So why should we trust research conclusions?

The answer seems to be that *trustworthiness* must be shown. This occurs at several levels. The raw data, the analytic methods, the analysts, and the organizations employing the analysts must all be shown to be worthy of being trusted. With these as necessary conditions for trusting conclusions of data analysis, we can seek ways to verify trustworthiness.

A key aspect of data trustworthiness is its provenance.^23^ Do we know where the data have come from and has the source proven reliable and trustworthy in the past?

Properly identified provenance is critical. And this applies more widely—to newspaper articles and blogs. Solidly established provenance will go some way toward tackling false news and fake facts. If such news items do not tell you where they got the data, and enable you to track back to the source yourself, you should be suspicious: what are they trying to hide? This does not necessarily mean that you *have to* track back—life is too short—but you should be able to. Blockchain technology may be particularly valuable here, giving an immutable record leading you back to the origin of the data—although since particular value often arises from merging multiple sources of data, this may not be trivial.

In general, quality is easier to monitor if the data come from a single source, and matching of data is a complex and risk-prone operation.^24^ Moreover, data quality depends on the objective: “quality” is not an absolute, it can be high for one purpose and low for another.^11^ Worse still, quality is not static in this sense that in many situations, data become less useful for answering specific questions unless regularly updated.

The notion of open data is also relevant here. If data can be open, without compromising other ethical principles, then provenance assurance is stronger: more eyes mean fewer lies, and fewer mistakes.

We have already commented that numbers alone do not constitute data: data are a combination of the numbers and what they mean. Comprehensive metadata are also vital. If you cannot understand the data, you should not trust it.

Adequacy of metadata leads us to adequacy of description of methods. And in just the same way that trust requires one to understand the data and be able to track them back to origin, so trust requires that an analysis should be reproducible. This is distinct from replicability, and the (often misguided) controversy about whether statistical results can be reproduced, but simply a matter of whether accounts give sufficient detail to enable one to repeat them. If they do not, we should ask, “what are they not telling us?” Have they analyzed only part of the data? Have they omitted critical confounding variables? And a host of related questions that statisticians are familiar with.

A distortion that often occurs with “big data” is when the data are exhaust from some operation. Then, one has to adapt one's question to the available data, and this might not be a good match for the question of interest.^11^ A method cannot be trusted if it is being used to answer the wrong question. A variant of this can lead to algorithms that are intrinsically discriminatory because the data on which they are trained do not match the population distribution.

A particularly colorful variant of this is manifest in the concept of a “horse” in machine learning. A horse is “a system appearing capable of a remarkable human feat … but actually working by using irrelevant characteristics.”^25^ Examples of the potential dangers are widespread, from music information retrieval systems to image recognition.

The core underlying problem with horses arises from the distinction between data-driven and theory-driven models.^26–30^ A theory-driven model is based on some underlying theory of mechanism, describing how variables might be related (e.g., Newton's theory of gravitation). A data-driven model is based purely on observed relationships within the data. Data-driven analysis can lead to great discoveries. However, it can also lead to great mistakes as it picks up on relationships in the data arising by chance, due to inappropriate sample selection, or for some other reason. David Haig's play *Pressure* gives a nice illustration of this. The play describes the uncertainty over weather conditions at the time of the planned D-Day landings in northern France during the Second World War. Meteorologist Irving Krick bases his forecasts on similarities between today's weather conditions and previous conditions, arguing that very similar conditions lead to very similar outcomes. A purely data-driven analysis. James Stagg, on the contrary, argues that high-altitude wind patterns should also be taken into account. A theory-based analysis.

A more general message to take away from the model-type distinction is that one should always remember that a model is just a model. It is not the underlying reality (if there is one) being modeled. At best, a model is necessarily a simplification and abstraction, with the world always being more complicated, often in unsuspected ways. Trustworthiness depends on the veracity of the model as a representation of the relevant aspects of the world.

Issues of trust in the data and trust in the methods often conceal deep ethical considerations, which are not so easily resolved. One example arises in discrimination, where inconsistent definitions exist.^31^ Another example occurs in dynamic pricing and the extent to which it is legitimate to adjust prices based on algorithmic estimates of how much a customer can pay.

Trust in the data and trust in the methods get us a large part of the way to our destination. However, there is also a human element. We need to trust the analysts—to be competent and to be honest. There are plenty of examples of fraud, scientific and otherwise, and of elementary mistakes in understanding and application. Training and accreditation are clearly important.

## Privacy and Confidentiality

The fact that privacy will play a central role in considerations of data ethics is indicated by its appearance in Article 12 of the *Universal Declaration of Human Rights* (1948), which begins “No one shall be subjected to arbitrary interference with his [sic] privacy ….” However, privacy can be defined in various ways. Fifty years ago, Westin^32^ wrote “few values so fundamental to society as privacy have been left so undefined in social theory or have been the subject of such vague and confused writing by social scientists” and more recently Mulligan et al.^33^ describe privacy as “an essentially contested concept.” This is based on the argument by Gallie^34^ that it is false to suppose that concepts are either clear or confused, but that there are other concepts “the proper use of which inevitably involves endless disputes about their proper uses on the part of their users.”

Among other definitions are that privacy is the right to be left alone and not be bothered, that it is the right to be protected from government intrusion, and that it is the power to selectively reveal oneself to the world. This last allows one to determine how one is perceived, suggesting that privacy control is a key aspect of one's identity. Indeed, we all present different faces to people we encounter in different circumstances. However, in the context of data, Mulligan et al.^33^ go so far as to say that “Informational self-determination [that is, the capacity of the individual to determine in principle the disclosure and use of his/her personal data] can hardly be considered a sufficient objective, nor individual control a sufficient mechanism, for protecting privacy in the face of this new class of technologies and attendant threats.”

However, one defines it, like data, privacy is not an absolute. It exists only relative to the person from whom we wish to keep something hidden. In general, privacy is dependent on the context and relationship between giver and receiver of data, and also on the use to which the data will be put. Indeed, public attitudes to privacy can change rapidly, often in response to reports of data leaks. Such fluctuations can have an adverse effect on the value of data sets if they allow people to opt in or opt out of being included. The media scare stories are seldom balanced by the notion that sharing (some kinds of) data might be seen as a moral responsibility. The UK experience over *care.data* is a case study of these conflicting forces.^35^ Neither are perceptions of privacy the same everywhere around the world. Appendix B of Waldo et al.^36^ explores similarities and differences between nations. One example is the difference between the United Kingdom and Scandinavia in attitudes to identity cards.

Personal privacy runs up against the importance of using data to promote the wider good: sometimes it is unethical *not* to use available data. A simple example would be revealing that someone has a highly contagious and inevitably fatal disease, but there are many less extreme examples. In general, as always, it is necessary to strike a balance between different kinds of risks and different kinds of gains. Striking such a balance would be straightforward enough if the risks and gains were clear and measurable. However, in real life this is seldom the case. Any given action might benefit one group or individual while harming others. Moreover, things change and, worse, unexpected consequences are all too common. We have already mentioned further complications arising from excessive caution in response to some of the risks (e.g., the ADRN), or excessive enthusiasm in the face of possible gains. In general, the more a company/organization/individual knows about you, or as we might say the more it invades your privacy, the more it can identify potential benefits for you. Again a simple example would be that a doctor who knows your medical history and symptoms would be much more useful to you than one who did not.

Modern data technology often tips the balance in one direction or another. A classic case is the creation of Voter Vault in the 1960s by the Republican Party in the United States, but the idea goes back for many years before that. The Voter Vault was a database of voters, containing details of their beliefs, attitudes, and opinions. Such databases mean that a tipping point has been reached. Whereas previously what mattered was what the voters knew about the candidates, what matters now is what the candidates know about the voters. With such knowledge, they can target undecided voters, orient their pitch on matters where they agree with the specific voter, and in general optimize the way they approach voters. At the time of writing, these ideas have hit the press, with the furore over Cambridge Analytica and Facebook data allegedly having been used to sway recent elections (e.g., Cadwalladr and Graham-Harrison^37^ and Scott^38^).

Of course, the use of such databases for electoral purposes is just a different manifestation of their use in other contexts. “Loyalty cards” do not really indicate loyalty to one particular retailer—people typically hold multiple such cards, for different retailers. Rather what they do is enable organizations to capture data about people, their preferences, their habits, and so on. And in return, customers are given reduced price offers, coupons, and so on. For retailers at least, it is not so much exploitation as a deal.

Such a balance is also manifest in what Wittes and Liu^39^ call the *privacy paradox*. They point out that the privacy balance is rather more nuanced than is typically understood. They say, “our behaviour as consumers is often exquisitely attuned to the reality that the march of technological development is not—contrary to the assumption that so dominates the privacy literature—simply robbing us of our privacy in exchange for convenience. Rather, technologies often offer privacy with one hand while creating privacy risks with the other, and consumers choose whether or not to use these technologies based, in part, on whether they value more the privacy given or the privacy taken away.” They give the colorful example of how nowadays one can access pornography through the web, avoiding the public nature of buying pornographic magazines in a newsagent. Indeed, as sexual predators and fraudsters have found, the Internet enhances privacy.

Concepts of personal data and privacy are often supposed to hinge on “personally identifiable information,” such as names, addresses, usernames, passwords, account codes, e-mail addresses, and ID numbers. The notion is that if such information is removed from a database, then the entries are anonymized, meaning that they cannot be matched to their owners. This, however, is often not true. With sufficient effort, and with the capability to match databases, it is often possible to reidentify supposedly anonymized records—for an example, see Narayanan and Shmatikov.^40^ In general, I doubt that the concept of “identifying information” is meaningful. *All* information is “identifying” if used in conjunction with other suitable material.

Overall, reliance on blanket high-level rules for privacy protection often fails because it does not allow for changing circumstances, new data sets appearing, and the linking of data. However, other strategies have been devised for carrying out analyses without divulging identities under various circumstances. These include secure multiparty computation, trusted third-party systems, and homomorphic encryption.

## Conclusion

The discussion above has covered the nature of data, especially personal data, whether the notion of ownership is meaningful, consent and purpose, trustworthiness of data as well as of algorithms and of those using the data, and matters of privacy and confidentiality. None of these topics is straightforward in a data context: all have nuances and even apparent contradictions. A primary source of the complexities is the fact that the use to which data might be put is fundamentally unknowable—but this is the source of the very power of data science, that some marvelous new and perhaps even unsuspected insight might emerge from the perspicacious analysis of data.

In striving to strike an appropriate balance between benefit and risk, we need to be clear about who benefits and who incurs the risk. If these are borne by different actors, imbalances can occur—and indeed ethical disasters can result. The question is not a trivial one, with a familiar example being the fact that benefit and risk might refer to individuals or to groups—and it is entirely possible that individual and group benefits might work in different directions. As to public benefit, complications arise such as a corporation claiming that promoting its product benefits the public, privacy intrusiveness preventing wrongdoing, and indeed social credit systems providing a larger scale benefit. Unfortunately, the law of unexpected consequences lurks behind everything.

Different bodies have taken different concepts as the core to data ethics, although they have closely related flavors. The European Data Protection Supervisor takes human dignity as a central motivating force: “The EDPS considers that better respect for, and the safeguarding of, human dignity could be the counterweight to the pervasive surveillance and asymmetry of power which now confronts the individual. *It should be at the heart of a new digital ethics*”^41^ (my italics). The Data Governance Working Group takes human flourishing as “the overarching principle that should guide the development of systems of data governance.”^42^

However, ethical principles are not necessarily universal. We might note, for example, that the United Kingdom is more bound by ethical considerations of data collection and use than some other countries. This could put us at a technological disadvantage. “Ethical drag” might mean a lack of agility in the face of evolving data technologies. Indeed, in the United Kingdom and many other countries, there is already a network of laws guiding and constraining what may be done with data—see, for example, the joint Royal Society/British Academy *Data Governance Landscape Review.*^43^ In the United Kingdom, these include the following:

- The GDPR, discussed above.
- The Freedom of Information Act.^44^ Its fundamental aim is to promote trust in government by providing the public with access to data held by public authorities. It requires such bodies to publish information about their activities and obliges them to provide information to members of the public when asked. “Public authorities” are government departments, local authorities, the National Health Service, state schools, and police forces. This Act does not enable people to access their own personal information, but this can be requested via the DPA and later the GDPR as described above.
- The Digital Economy Act^45,46^ is a broad piece of legislation concerned with electronic communications, data sharing, direct marketing, and a wide range of other issues. For us, the relevant section is Part 5, on Digital Government. This covers topics such as disclosure of information to public bodies and utilities, confidentiality of personal information, debt, fraud, and the sharing of data for research purposes and to the UK Statistics Authority (“the Board”).

And of course there are penalties for governance breaches: membership of bodies can be revoked for breaches of ethical guidelines, research funding might be restricted, and as we noted above, the GDPR specifies that the fines can be up to 4% of global annual turnover.

The GDPR also says (Recital (65)): “A data subject should have the right to have personal data concerning him or her rectified and a ‘right to be forgotten’ where the retention of such data infringes this Regulation or Union or Member State law to which the controller is subject.” However, this, as with any system which allows individuals to decide whether they should be included in the database (whether they are opt-in or opt-out systems), leads to a serious risk of selection bias, with the possible consequence that analyses are invalid—and decisions and actions suboptimal or worse. In a similar vein, data minimization is feasible when specific projects are in mind, but not in general. Indeed, in a sense, the idea of data minimization is contrary to the notion that data might be used in unsuspected ways (especially if merged with other data sources) to yield value. That is, in a sense it is counter to the very promise of the data revolution, possibly threatening to undercut it and render it ineffective.

It is inevitable that unintended consequences may emerge from today's (and tomorrow's) elaborate data ecosystem. We are all very much aware of adverse consequences arising from industrialization, including such things as climate change and plastic pollution of the seas. For data, as the Data Governance Working Group put it^42^ “Tangible harms can include detriment to health, financial loss, or discriminatory treatment. Intangible harms could arise as a result of exclusion from services, facilities or opportunities, or the fear that personally identifiable data may fall into the hands of those who exploit them unfairly. Although these harms are often difficult to detect and to quantify, they are nevertheless real, and often the cause of substantial distress and anxiety.” We must recognize that studies aimed at research—perhaps summarizing data to determine some overall property or perhaps to identify individuals with specific characteristics—will have slightly different requirements from operational studies aimed at decision-making (which may be in real time).

Other domains typically have checklists for ethical matters, often in the shape of forms that need to be completed and approved by an ethics committee before work can proceed. The same sort of strategy is appropriate for data ethics. Particular topics that need to be covered include the need to:

- identify which ethics body has oversight of the work;
- be aware of institutional policies and procedures;
- be aware of national regulations and laws (e.g., regarding privacy, consent, discrimination, and requirement to explain analysis);
- keep record of how data are modified and manipulated;
- understand the origin of the data (provenance);
- treat the metadata as rigorously as the data;
- have an explicit data management plan;
- store data securely;
- determine for how long the data must be kept;
- specify who has access to the data;
- ensure that appropriate statistical, machine learning, data mining, and so on tools are being used;
- have systems in place that allow data to be corrected (and deleted if necessary); and
- be clear about the benefits of the analysis, and who derives the benefits.

We remarked above that the notion that the data revolution is heading into a new “normal” is mistaken. In particular, this means that once we have overcome the data ethical challenges facing us today, we will be presented with new ones tomorrow. More than this, however, we will also find that the challenges we thought we had adequately met have sometimes been reopened, with new kinds of data, new modes of collection, and new opportunities for analysis. To take a simple and clear example: if quantum computation becomes a practical and useful reality, it will lead to a massive further change in our analytic capabilities, not to mention possibly compromising the encryption systems on which the finance and other sectors depend. All of this means that a code of ethics should not be regarded as set in stone. Especially in a field developing as fast as data technology, it is likely that the future will present challenges that are currently unsuspected. A data ethics code and guidelines should be re-examined every few years.

## Abbreviations Used

ADRN: Administrative Data Research Network
DPA: Data Protection Act
EU: European Union
GDPR: General Data Protection Regulation
